# First Complete Genome Sequence of the Dutch Veterinary *Coxiella burnetii* Strain NL3262, Originating from the Largest Global Q Fever Outbreak, and Draft Genome Sequence of Its Epidemiologically Linked Chronic Human Isolate NLhu3345937

**DOI:** 10.1128/genomeA.00245-16

**Published:** 2016-04-21

**Authors:** Runa Kuley, Hilde E. Smith, Ingmar Janse, Frank L. Harders, Frank Baas, Elio Schijlen, Marrigje H. Nabuurs-Franssen, Mari A. Smits, Hendrik I. J. Roest, Alex Bossers

**Affiliations:** a Department of Infection Biology, Central Veterinary Institute, part of Wageningen UR, Lelystad, The Netherlands; b Department of Bacteriology and Epidemiology, Central Veterinary Institute, part of Wageningen UR, Lelystad, The Netherlands; c Host Microbe Interactomics, Wageningen University, Wageningen, The Netherlands; d National Institute for Public Health and the Environment, Bilthoven, The Netherlands; e Department of Genome Analysis, Amsterdam Medical Centre, Amsterdam, The Netherlands; f Department of Bioscience, Plant Research International, part of Wageningen UR, Wageningen, The Netherlands; g Department of Medical Microbiology, Canisius-Wilhelmina Hospital, Nijmegen, The Netherlands

## Abstract

The largest global Q fever outbreak occurred in The Netherlands during 2007 to 2010. Goats and sheep were identified as the major sources of disease. Here, we report the first complete genome sequence of *Coxiella burnetii* goat outbreak strain NL3262 and that of an epidemiologically linked chronic human strain, both having the outbreak-related *CbNL01* multilocus variable-number tandem-repeat analysis (MLVA) genotype.

## GENOME ANNOUNCEMENT

Q fever is a zoonotic disease caused by *Coxiella burnetii*. Starting in 2007, The Netherlands has been confronted with the largest global Q fever outbreak ever, involving 4,026 human cases. Based on epidemiological and genotyping studies, dairy goats and sheep were identified as the main sources of the human Q fever outbreak. Special attention was given to the Dutch outbreak-specific strain of the *CbNL01* multilocus variable-number tandem-repeat analysis (MLVA) genotype, which was identified in abortive dairy goats and in humans (1–6). The current project was performed with *C. burnetii* strains of this predominant genotype isolated from an aborted goat placenta (1, 7) and from a heart valve of a chronic Q fever patient during the outbreak period. Genome determination of the veterinary NL3262 strain is crucial for understanding the large outbreak and biology of this highly virulent strain. The genomes of virulent veterinary (NL3262) and related human (NLhu3345937) outbreak strains were sequenced, and their reconstructed genomes were compared to the examine similarities and differences in their genome structures.

Strains NL3262 and NLhu3345937 were cultivated axenically in acidified citrate cysteine medium (ACCM-2) and BGM cells, respectively (7, 8). Genomic DNA was isolated using the phenol-chloroform method (9). A prior DNase treatment was performed to the bacterial pellet of NLhu3345937 to eliminate most host-derived DNA. The genome of NL3262 was *de novo* reconstructed from mixed assemblies using a combination of PacBio RS, Roche 454XL, and Illumina PE250 MiSeq reads. Reconstructed genomes were improved with different Illumina read sets, with a total average coverage of 600× using Pilon-1.8 (10). The NLhu3345937 genome was *de novo* reconstructed with Illumina PE250 MiSeq reads using SPAdes-3.6.2 (11), for a total average coverage of 285×.

The complete NL3262 sequence and draft genome sequence of NLhu3345937 contain 2,093,477 and 2,088,566 bp, with G+C contents of 42.9 and 42.6%, respectively. Genome comparisons of NL3262, NLhu3345937, and the draft NL-Limburg outbreak strains (12) using Nucmer (13), MUMi-BioNJ tree (14), and visualized using Artemis Comparison Tool (ACT) (15) show that these strains are closely related, with differences mainly based on single-base-pair mutations. Our veterinary and human strains both contain the ~37-kb pQpH1 plasmid similar to that in the reference NM-RSA493 strain. Genome annotation of NL3262 by NCBI Prokaryotic Genome Annotation Pipeline (http://www.ncbi.nlm.nih.gov/genome/annotation_prok) showed 2,101 coding sequences (CDSs), 180 pseudogenes, 42 tRNAs, 3 rRNAs, and 1 noncoding RNA (ncRNA). Compared to *C. burnetii* NM (accession no. NC_002971.3), the majority of genes (84%) are orthologs found in both strains, with only 16% of the genes specific to NL3262 comprising mainly hypothetical products. Genome reconstruction was cumbersome between different passages of each strain due to high genome complexity, as described before for other isolates (many transposons, repetitive IS*1111* elements, and rearrangements) (16). The observed high similarity between veterinary (NL3262) and human (NLhu3345937 and NL-Limburg) strains confirms the previous epidemiological and genotypic studies linking the goat and human infections during the Q fever outbreak in The Netherlands. A detailed comparative genome analysis of several sequenced *C. burnetii* isolates from different origins is ongoing.

### Nucleotide sequence accession numbers.

*C. burnetii* NL3262 and NLhu3345937 genome and plasmid sequences have been deposited in GenBank under accession numbers CP013667, CP013668, CP014354, and CP014355.
